# Knowledge, Attitudes, and Practices of Avian Influenza, Poultry Workers, Italy

**DOI:** 10.3201/eid1211.060671

**Published:** 2006-11

**Authors:** Rossella Abbate, Gabriella Di Giuseppe, Paolo Marinelli, Italo F. Angelillo

**Affiliations:** * Second University of Naples, Naples, Italy;; † University of Catanzaro Magna Graecia Medical School, Catanzaro, Italy

**Keywords:** Avian influenza, attitude, behavior, Italy, knowledge, poultry workers, prevention, practices, dispatch

## Abstract

We asked Italian poultry workers about knowledge, attitudes, and practices regarding avian influenza. It was perceived to be a low occupational hazard, and wearing protective equipment and handwashing were not routine practices. Knowledge of transmission and preventive measures should be improved. Employers and health professionals should provide more effective information.

Infection of poultry with influenza A (subtype H5N1) virus is responsible for outbreaks in birds and a human case-fatality rate of 58% (*1*). The most likely means of transmission is from infected birds to humans and from the environment to humans, but evidence for human-to-human transmission is limited (*2*). This virus can be transmitted if a person has direct contact with infected poultry or surfaces and objects contaminated by poultry droppings. Two epidemics caused by avian influenza virus H5 and H7 subtypes occurred in poultry in Italy from 1997 through 2001. A plan was recently developed for adequate response to influenza pandemics, and farmers, veterinarians, and healthcare workers have been educated about diagnosing, detecting, and preventing the spread of avian influenza (*3*).

Workers in the poultry industry, who commonly have contact with live, sick, or dying poultry, are at high risk for avian influenza. These workers are at increased risk because of food handling and preparation of raw poultry meat and products. Concern exists that avian influenza could be transmitted from uncooked birds or bird products to humans (*4**,**5*). This study evaluated knowledge, attitudes, and infection control practices of poultry workers in Italy regarding avian influenza.

## The Study

**Figure A1:**
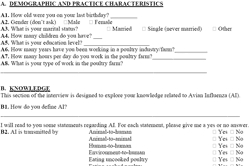
Survey questions, available in two Adobe Acrobat files: Download English PDF, Download Italian PDF.

A total of 284 poultry workers at 110 poultry farms throughout the Campania region of Italy were recruited into the study from December 2005 through March 2006. The workers were interviewed confidentially in their workplace regarding demographics, work activity, knowledge of transmission and prevention of avian influenza, attitudes toward this disease, compliance with precautions at work, and sources of information (Figure A1). Multiple logistic regression analysis with adjusted odds ratios and 95% confidence intervals and multiple linear regression analysis with adjusted β coefficients were performed with Stata software (*6*).

A total of 257 poultry workers were interviewed (response rate 90.5%). Average age was 43 years (range 19–75 years), average duration of work activity was 18 years, and median number of daily exposures to breeder animals was 18,500. One third of the workers had a high school or college education.

Of the 257 workers, 63.8% correctly defined avian influenza as a contagious infection caused by a virus that can affect all species of birds (Table 1), and 21.8%–81.7% knew that avian influenza can be transmitted by touching uncooked eggs or infected animals. Nearly all workers identified poultry and wild birds as common vectors. Most knew that poultry workers had a high risk of being infected and that butchers and veterinarians had a lower risk. Only 22.6% provided a correct definition of this disease and knew routes and vectors of transmission.

**Table 1:** Knowledge of avian influenza among 257 poultry workers, Italy

Variable	Correctly answered, no. (%)
Definition (contagious infection caused by virus that can affect all species of birds)	164 (63.8)
Modes of transmission	
Animal to human	210 (81.7)
Animal to animal	206 (80.2)
Environment to human	153 (59.5)
Eating uncooked poultry	149 (58)
Eating uncooked eggs	102 (39.7)
Touching uncooked poultry	88 (34.2)
Touching uncooked eggs	56 (21.8)
Touching wild birds	246 (95.7)
Touching poultry	232 (90.3)
Touching saliva, nasal secretions, feces, and fomites of infected birds	167 (65)
Risk groups	
Poultry workers	194 (75.5)
Butchers	134 (52.1)
Veterinarians	108 (42)
Use of preventive measures	
Face mask	176 (68.5)
Gloves	158 (61.5)
Outer garments	157 (61.1)
Boots or boot covers	144 (56)
Eye protection	111 (43.2)
Handwashing with soap and water	161 (62.7)

Knowledge was greater in persons with more education, those who worked a longer time, those who believed they were at high risk of contracting avian influenza, and those who needed information (Table 2). With respect to identifying measures that protect poultry workers from exposure to avian influenza, correct responses ranged from 34.2% for all protective measures to 43.2% for eye protection and 68.5% for face masks. Greater knowledge was observed in those who received information from health professionals and employers, those who believed they were at high risk, and those who worked only with poultry (Table 2).

**Table 2:** Logistic and linear regression models results of knowledge, attitudes, and practices of avian influenza among 257 poultry workers, Italy*

Variable	OR		95% CI		p value		
Model 1: General knowledge about avian influenza; log likelihood –124.45, χ^2^ 25.58, df 6, p = 0.0003							
Years of working activity	1.03		1.01–1.06	0.015			
Need of additional information	2.59		1.17–5.72	0.019			
Perception of risk for avian influenza	1.13		1.01–1.26	0.037			
Education level	1.63		1.02–2.61	0.042			
Avian influenza is a serious and preventable disease	1.44		0.72–2.88	0.31			
No. breeder animals exposed to per day	0.87		0.65–1.16	0.33			
Model 2: Knowledge of all measures that protect poultry workers from exposure to avian influenza; log likelihood –142.95, χ^2^ 44.40, df 7, p < 0.0001							
Health professionals and employers as sources of information	3.62		1.99–6.59	<0.001			
Perception of risk for avian influenza	1.19		1.07–1.34	0.002			
Working with poultry and eggs	1.0†		–	–			
Working with only poultry	2.11		1.17–3.81	0.013			
Education level	1.37		0.90–2.08	0.14			
General knowledge of avian influenza	1.44		0.74–2.8	0.28			
No. breeder animals exposed to per day	0.88		0.68–1.14	0.33			
Hours worked per day	1.05		0.95–1.14	0.35			
Model 3: Modification of working habits in the past 3 mo; log likelihood –119.48, χ^2^ 42.72, df 10, p < 0.0001							
Health professionals and employers as sources of information		0.34	0.17–0.69	0.003			
Marital status		0.31	0.13–0.76	0.01			
Age		0.96	0.92–0.99	0.018			
Avian influenza is a serious and preventable disease		2.29	1.09–4.76	0.028			
General knowledge of avian influenza		2.11	1.03–4.31	0.041			
Poultry workers are a risk group		2.30	0.94–5.65	0.07			
Perception of risk for avian influenza		1.09	0.97–1.22	0.15			
Working with poultry and eggs		1.0†	–	–			
Working with only poultry		0.64	0.03–1.34	0.24			
Sex		1.45	0.73–2.86	0.29			
Education level		1.32	0.78–2.22	0.3			
Model 4: Preventive measures behavior; log likelihood –119.36, χ^2^ 45.26, df 4, p < 0.0001							
Knowledge of preventive measures		5.95	3.06–11.56	<0.001			
Working with poultry and eggs		1.0†	–	–			
Working with only eggs		0.28	0.11–0.73	0.009			
General knowledge of avian influenza		1.48	0.72–3.02	0.28			
Health professionals and employers as sources of information		1.37	0.70–2.69	0.37			
Variable		β coefficient	t			p value
Model 5: Perception of risk for avian influenza; F(7,249) = 8.25, p < 0.0001, R^2^ 18.8%, adjusted R^2^ 16.6%						
Working with poultry and eggs		–†	–			–
Working with only poultry		–1.72	–5.0			<0.001
Hours worked per day		-0.19	-3.74			<0.001
Need additional information		1.16	3.56			<0.001
Media and television as sources of information		2.02	3.36			0.001
Knowledge of preventive measures		0.77	2.36			0.019
No. children		-0.21	-1.56			0.12
Sex		-0.32	-0.9			0.37
Constant		3.42				

*OR, odds ratio; CI, confidence interval; df, degrees of freedom. †Reference category.

Most poultry workers believed that avian influenza was a serious (69.7%) but preventable (70.8%) disease. Mean total scores (scale of 1 to 10) for perceived risk of contracting avian influenza during work activity and for co-workers and family members were 3.2 and 3.1, respectively, which indicated low-risk perception. Only 4.3% showed great concern about risk. Respondents who were more likely to believe that they were at high risk worked fewer hours, knew protective measures for exposure to avian influenza, had received information from the mass media, and needed information. Workers who were exposed only to poultry were less likely to perceive risk (Table 2).

A total of 23.7% reported that in the past 3 months they had modified their work habits because of fear of contracting avian influenza. Those more likely to modify their behavior were younger, married, had more knowledge of avian influenza, believed that it was a serious but preventable disease, and received information from sources other than health professionals and employers (Table 2).

Regarding compliance with precautions to avoid spreading virus through food while working, 59.9% routinely washed their hands and disinfected surfaces and utensils that had been in contact with raw meat. Wearing personal protective equipment was not a routine practice because 82.9% always wore outer garments, 82.9% wore boots or protective boot covers, 59.9% wore gloves, 59.9% wore face masks, 24.5% wore eye protection, and 87.9% washed their hands. A total of 24.1% always wore protective clothing and washed their hands; these practices were more common in poultry workers who knew that these measures were protective and less common by workers who handled only eggs (Table 2).

All poultry workers had received information regarding avian influenza. The most common sources were mass media (91.8%), health professionals (47.5%), and employers (6.2%); 62.3% wanted more information.

## Conclusions

Knowledge of avian influenza can be improved, as shown in a study of consumers (G. Di Giuseppe et al., unpub. data). We expected to find more knowledge in educated poultry workers, especially in how to identify potential animal cases and minimize risk for transmission. However, our observations indicate that information is not correctly disseminated because those who receive information from the mass media and who need information were more likely to have a perception of high risk. Therefore, tailored educational programs, including booklets and seminars, could be beneficial in improving self-risk assessment of poultry workers.

Two thirds of poultry workers believed that avian influenza is a serious and preventable disease, but the study showed a perception of low risk of contracting this disease at work because only 4.3% indicated strong concern, although the largest percentage correctly recognized that they are a risk group. Workers who handled only poultry were less likely to perceive a risk than those who handled other products.

Studies have identified direct exposure to infected poultry as the primary risk factor in transmission of avian influenza virus to humans. A cohort study of poultry workers in Hong Kong showed that greater exposure to poultry was associated with antibody to H5 hemagglutinin (*4*). In Thailand, a case-control study showed that activities involving exposure to poultry were associated with influenza caused by H5N1 virus (*7*). In Vietnam, a population study in a rural area with outbreaks of highly pathogenic avian influenza showed a dose-response relationship between poultry exposure and illness (*8*).

Low adherence to the recommendations of the World Health Organization to avoid spread of avian influenza through food while working has been reported; use of protective clothing and handwashing is inadequate (*9*). In our study, there was a subset of workers who routinely followed guidelines because lower compliance was observed in those working only with eggs. Moreover, those who did not know precautions had a 6-fold greater risk for inconsistent adherence to preventive guidelines compared with those who identify them.

Improving knowledge of transmission and application of preventive measures is a useful public health strategy for reducing the effects of avian influenza in poultry workers. Employers and health professionals should work together to provide effective and coordinated information to these workers.
